# Crystal structure of 5-hy­droxy­methyl-2-meth­oxy­phenol

**DOI:** 10.1107/S205698901501230X

**Published:** 2015-07-04

**Authors:** Mubashir Hassan, Zaman Ashraf, Sung-Yum Seo, Daeyoung Kim, Sung Kwon Kang

**Affiliations:** a Department of Biology, College of Natural Sciences, Kongju National University, Gongju 314-701, Republic of Korea; b Department of Chemistry, Allama Iqbal Open University, Islamabad 44000, Pakistan; c Department of Chemistry, Chungnam National University, Daejeon 305-764, Republic of Korea

**Keywords:** crystal structure, alcoholic hy­droxy compounds, O—H⋯O hydrogen bonding

## Abstract

In the title compound, C_8_H_10_O_3_, the hy­droxy­methyl group is twisted by 74.51 (13)° from the plane of the benzene ring to which it is connected. By contrast, the benzene and meth­oxy groups are almost coplanar, making a dihedral angle of 4.0 (2)°. In the crystal, O—H⋯O hydrogen bonds link the mol­ecules into a three-dimensional network.

## Related literature   

For the background to alcoholic hy­droxy compounds and their applications, see: Patrick (2001 ▸); Yasohara *et al.* (2001 ▸); Rodríguez-Barrios & Gago (2004 ▸); Wu *et al.* (2008 ▸); Matteelli *et al.* (2010 ▸); Coimbra *et al.* (2010 ▸); Hans *et al.* (2010 ▸); Cordova *et al.* (2006 ▸). For the synthesis of derivatives of the title compound, see: Ashraf *et al.* (2014 ▸, 2015 ▸).
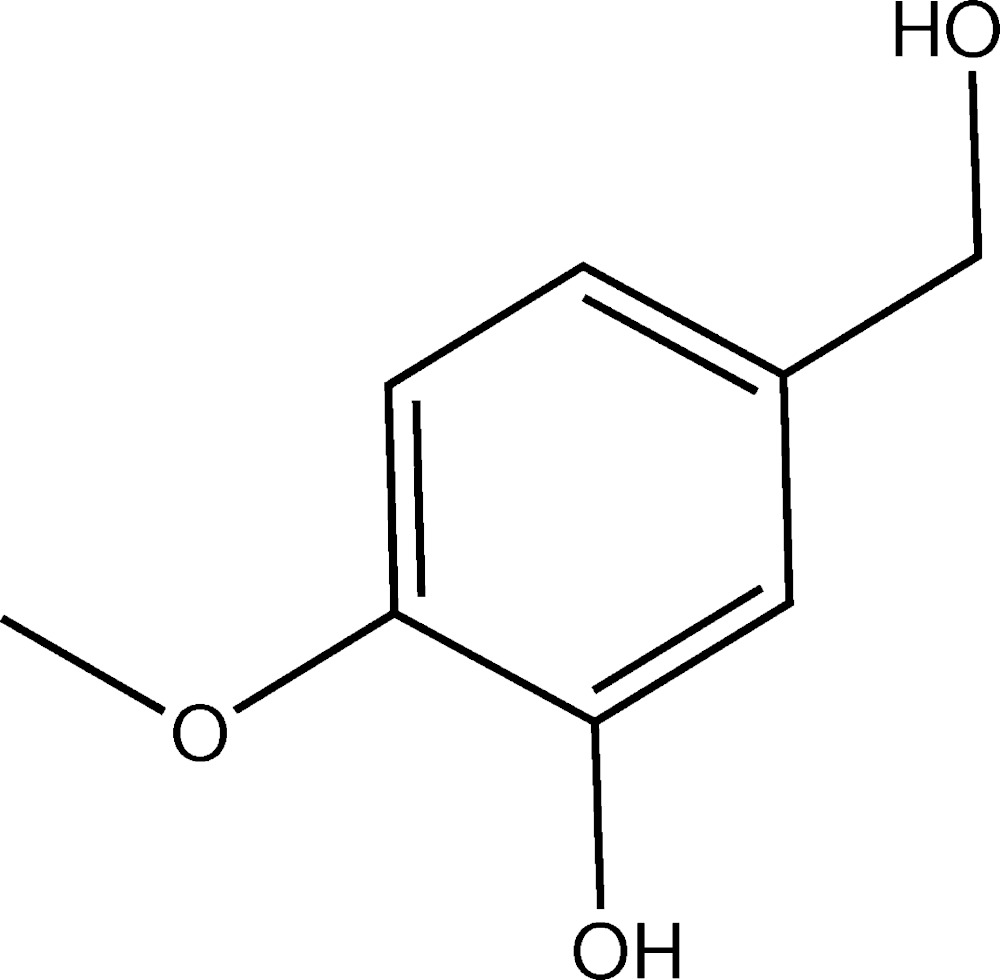


## Experimental   

### Crystal data  

C_8_H_10_O_3_
*M* *_r_* = 154.16
Orthorhombic, 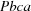

*a* = 15.011 (4) Å
*b* = 6.1354 (18) Å
*c* = 16.543 (5) Å
*V* = 1523.6 (7) Å^3^
*Z* = 8
Mo *K*α radiation
μ = 0.10 mm^−1^
*T* = 296 K
0.28 × 0.25 × 0.23 mm

### Data collection  

Bruker SMART CCD area-detector diffractometer
28952 measured reflections
1900 independent reflections
1530 reflections with *I* > 2σ(*I*)
*R*_int_ = 0.025

### Refinement  

*R*[*F* ^2^ > 2σ(*F* ^2^)] = 0.051
*wR*(*F* ^2^) = 0.149
*S* = 1.07
1900 reflections
108 parameters
H atoms treated by a mixture of independent and constrained refinement
Δρ_max_ = 0.38 e Å^−3^
Δρ_min_ = −0.42 e Å^−3^

### 

Data collection: *SMART* (Bruker, 2002 ▸); cell refinement: *SAINT* (Bruker, 2002 ▸); data reduction: *SAINT*; program(s) used to solve structure: *SHELXS2013* (Sheldrick, 2008 ▸); program(s) used to refine structure: *SHELXL2013* (Sheldrick, 2015 ▸); molecular graphics: *ORTEP-3 for Windows* (Farrugia, 2012 ▸); software used to prepare material for publication: *WinGX* (Farrugia, 2012 ▸).

## Supplementary Material

Crystal structure: contains datablock(s) global, I. DOI: 10.1107/S205698901501230X/tk5370sup1.cif

Structure factors: contains datablock(s) I. DOI: 10.1107/S205698901501230X/tk5370Isup2.hkl

Supporting information file. DOI: 10.1107/S205698901501230X/tk5370Isup3.cml

**Fig. 1.**
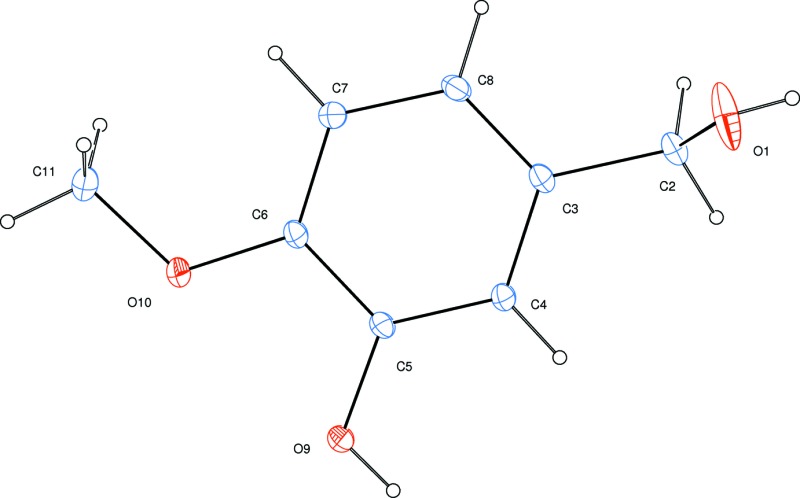
The molecular structure of the title compound, showing the atom-numbering scheme and 30% probability ellipsoids.

. DOI: 10.1107/S205698901501230X/tk5370fig1.tif
The mol­ecular structure of the title compound, showing the atom-numbering scheme and 30% probability ellipsoids.

**Fig. 2.**
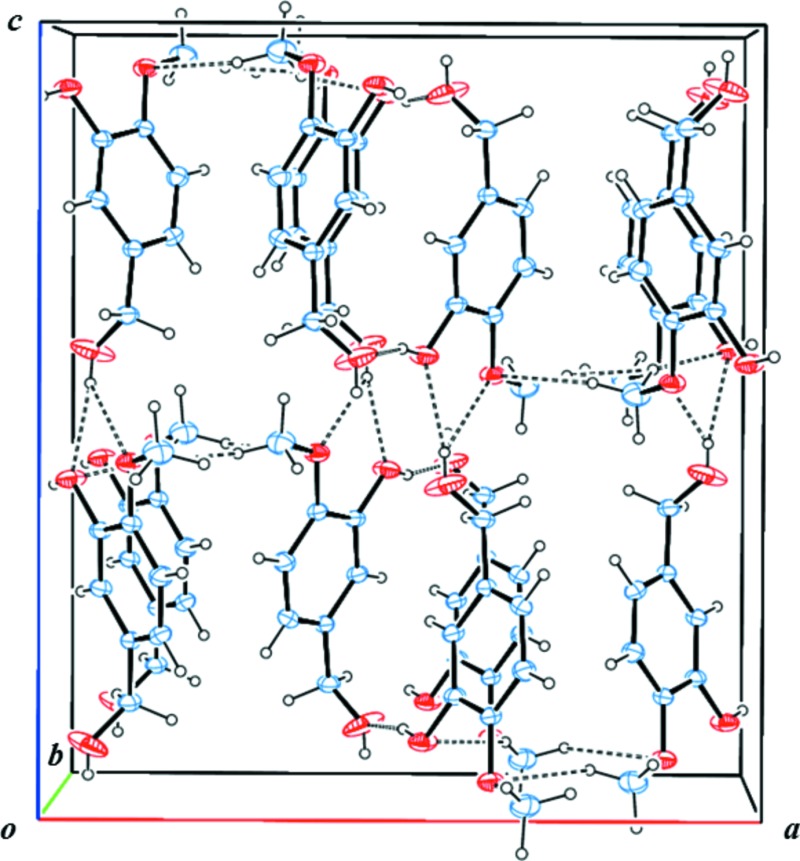
Part of the crystal structure of the title compound, showing the 3-D network of molecules linked by intermolecular O—H···O hydrogen bonds (dashed lines).

. DOI: 10.1107/S205698901501230X/tk5370fig2.tif
Part of the crystal structure of the title compound, showing the 3-D network of mol­ecules linked by inter­molecular O—H⋯O hydrogen bonds (dashed lines).

CCDC reference: 1409010

Additional supporting information:  crystallographic information; 3D view; checkCIF report

## Figures and Tables

**Table 1:** Hydrogen-bond geometry (, )

*D*H*A*	*D*H	H*A*	*D* *A*	*D*H*A*
O1H1O9^i^	0.74(4)	2.11(4)	2.773(2)	150(4)
O1H1O10^i^	0.74(4)	2.54(4)	3.152(2)	142(4)
O9H9O1^ii^	0.88(4)	1.78(4)	2.641(2)	163(3)

Symmetry codes: (i) 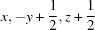; (ii) 
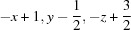
.

## supplementary crystallographic information

### S1. Chemical context

It has been identified that the presence of alcoholic, phenolic hydroxyl and amino groups are particularly important functionalities in biologically active compounds (Patrick, 2001). These important functionalities displayed biological activities because they can facilitate inter­actions with appropriate receptor molecules (Yasohara *et al.*, 2001). A new class of compounds having an alcoholic hydroxyl group showed high enzyme inhibitory potential and excellent permeation through a Caco-2 cell membrane (Rodríguez-Barrios & Gag, 2004). Some tertiary alcohol derivatives showed significant HIV-1 protease inhibitory activity (Wu *et al.*, 2008). Heterocyclic compounds such as di­aryl­quinolines having a quinolinic central nucleus and alcoholic –OH group at the side-chains exhibited anti-mycobacterial activity (Matteelli *et al.*, 2010). β-Amino alcohols are a class of compounds with a wide range of bioactivities, such as anti-plasmodial (Hans *et al.*, 2010), anti-leishmanial (Coimbra *et al.*, 2010) and anti-proliferative (Cordova *et al.*, 2006). The hy­droxy substituted benzoic acids and cinnamic acid analogues have been reported as mushroom tyrosinase inhibitors (Ashraf *et al.*, 2014; Ashraf *et al.*, 2015). Keeping in view the wide range of biological activities of hy­droxy­lated compounds, here we report the synthesis and crystal structure of the title compound, isolated as an inter­mediate. The title alcohol is a valuable starting material for the synthesis of hy­droxy substituted scaffolds.

### S2. Synthesis and crystallization

The title compound was synthesized by the reduction of isovanillin in the presence of sodium borohydride and methanol. The sodium borohydride reduction of aldehydes and ketones to the corresponding alcohols is a commonly used method in organic synthesis. Isovanillin was dissolved in methanol and then sodium borohydride was added at a slow rate to keep the reaction temperature below 25 °C. The excess of sodium borohydride was used to assure the completion of the reaction. After the completion of the reaction the product was obtained by acidic workup (86%, m.p. 135-137 °C). The title compound was crystallized as cubic crystals from a solution of ethyl acetate by slow evaporation.

### S3. Refinement

H atoms on OH groups were located in a difference Fourier map and refined freely [refined O—H distances = 0.74 (4) – 0.88 (4) Å]. The C-bound H atoms were positioned geometrically and refined using riding model, with d(C—H) = 0.93 – 0.97 Å, and with U_iso_(H) = 1.2*U*_eq_(C) for phenyl-H and methyl­ene-H and 1.5*U*_eq_(C) for methyl-H atoms.

### Crystal data

**Table:**

C_8_H_10_O_3_	*F*(000) = 656
*M**_r_* = 154.16	*D*_x_ = 1.344 Mg m^−^^3^
Orthorhombic, *P**b**c**a*	Mo *K*α radiation, λ = 0.71073 Å
Hall symbol: -P 2ac 2ab	Cell parameters from 7746 reflections
*a* = 15.011 (4) Å	θ = 2.5–28.2°
*b* = 6.1354 (18) Å	µ = 0.10 mm^−^^1^
*c* = 16.543 (5) Å	*T* = 296 K
*V* = 1523.6 (7) Å^3^	Block, brown
*Z* = 8	0.28 × 0.25 × 0.23 mm

### Data collection

**Table:**

Bruker SMART CCD area-detector diffractometer	*R*_int_ = 0.025
Radiation source: fine-focus sealed tube	θ_max_ = 28.4°, θ_min_ = 2.5°
φ and ω scans	*h* = −20→19
28952 measured reflections	*k* = −8→8
1900 independent reflections	*l* = −22→20
1530 reflections with *I* > 2σ(*I*)	

### Refinement

**Table:**

Refinement on *F*^2^	0 restraints
Least-squares matrix: full	Hydrogen site location: mixed
*R*[*F*^2^ > 2σ(*F*^2^)] = 0.051	H atoms treated by a mixture of independent and constrained refinement
*wR*(*F*^2^) = 0.149	*w* = 1/[σ^2^(*F*_o_^2^) + (0.0668*P*)^2^ + 0.6464*P*] where *P* = (*F*_o_^2^ + 2*F*_c_^2^)/3
*S* = 1.07	(Δ/σ)_max_ < 0.001
1900 reflections	Δρ_max_ = 0.38 e Å^−^^3^
108 parameters	Δρ_min_ = −0.42 e Å^−^^3^

### Special details

**Table:**

Geometry. All e.s.d.'s (except the e.s.d. in the dihedral angle between two l.s. planes) are estimated using the full covariance matrix. The cell e.s.d.'s are taken into account individually in the estimation of e.s.d.'s in distances, angles and torsion angles; correlations between e.s.d.'s in cell parameters are only used when they are defined by crystal symmetry. An approximate (isotropic) treatment of cell e.s.d.'s is used for estimating e.s.d.'s involving l.s. planes.

### Fractional atomic coordinates and isotropic or equivalent isotropic displacement parameters (Å^2^)

**Table:**

	*x*	*y*	*z*	*U*_iso_*/*U*_eq_	
O1	0.56019 (17)	0.3078 (3)	0.91644 (10)	0.1025 (9)	
H1	0.559 (2)	0.268 (6)	0.959 (2)	0.129 (13)*	
C2	0.61335 (11)	0.1812 (3)	0.86876 (9)	0.0462 (4)	
H2A	0.5895	0.0345	0.8663	0.055*	
H2B	0.6727	0.1737	0.8919	0.055*	
C3	0.61828 (10)	0.2752 (3)	0.78509 (9)	0.0383 (4)	
C4	0.57178 (10)	0.1775 (3)	0.72196 (8)	0.0388 (4)	
H4	0.5385	0.0524	0.7316	0.047*	
C5	0.57476 (10)	0.2651 (3)	0.64505 (8)	0.0375 (3)	
C6	0.62554 (10)	0.4516 (3)	0.62970 (9)	0.0368 (3)	
C7	0.67100 (11)	0.5504 (3)	0.69227 (10)	0.0452 (4)	
H7	0.7042	0.6757	0.6828	0.054*	
C8	0.66682 (11)	0.4616 (3)	0.76955 (9)	0.0454 (4)	
H8	0.6973	0.5292	0.8116	0.054*	
O9	0.52924 (10)	0.1784 (2)	0.58164 (7)	0.0554 (4)	
H9	0.498 (2)	0.060 (6)	0.5924 (18)	0.124 (12)*	
O10	0.62581 (8)	0.5189 (2)	0.55089 (7)	0.0472 (3)	
C11	0.67256 (15)	0.7139 (3)	0.53259 (12)	0.0588 (5)	
H11A	0.6682	0.7433	0.4757	0.088*	
H11B	0.7341	0.6976	0.5473	0.088*	
H11C	0.647	0.8328	0.5624	0.088*	

### Atomic displacement parameters (Å^2^)

**Table:**

	*U*^11^	*U*^22^	*U*^33^	*U*^12^	*U*^13^	*U*^23^
O1	0.1593 (19)	0.1051 (14)	0.0432 (8)	0.0824 (14)	0.0458 (10)	0.0312 (9)
C2	0.0512 (9)	0.0580 (10)	0.0293 (7)	0.0082 (7)	−0.0009 (6)	0.0039 (7)
C3	0.0371 (7)	0.0506 (9)	0.0272 (7)	0.0057 (6)	0.0004 (5)	0.0000 (6)
C4	0.0443 (8)	0.0425 (8)	0.0296 (7)	−0.0036 (6)	0.0043 (6)	−0.0003 (6)
C5	0.0429 (7)	0.0434 (8)	0.0263 (7)	−0.0021 (6)	0.0013 (5)	−0.0039 (6)
C6	0.0395 (7)	0.0431 (8)	0.0277 (7)	0.0011 (6)	0.0025 (5)	0.0014 (6)
C7	0.0469 (8)	0.0503 (9)	0.0384 (8)	−0.0110 (7)	−0.0005 (6)	−0.0007 (7)
C8	0.0443 (8)	0.0590 (10)	0.0327 (8)	−0.0063 (7)	−0.0063 (6)	−0.0050 (7)
O9	0.0752 (9)	0.0631 (8)	0.0279 (6)	−0.0280 (7)	−0.0037 (5)	−0.0024 (5)
O10	0.0610 (7)	0.0505 (7)	0.0302 (6)	−0.0110 (5)	0.0009 (5)	0.0060 (5)
C11	0.0761 (13)	0.0528 (11)	0.0476 (10)	−0.0137 (9)	0.0028 (9)	0.0127 (8)

### Geometric parameters (Å, º)

**Table:**

O1—C2	1.365 (2)	C6—O10	1.3677 (18)
O1—H1	0.74 (4)	C6—C7	1.380 (2)
C2—C3	1.501 (2)	C7—C8	1.391 (2)
C2—H2A	0.97	C7—H7	0.93
C2—H2B	0.97	C8—H8	0.93
C3—C8	1.380 (2)	O9—H9	0.88 (4)
C3—C4	1.392 (2)	O10—C11	1.420 (2)
C4—C5	1.382 (2)	C11—H11A	0.96
C4—H4	0.93	C11—H11B	0.96
C5—O9	1.3601 (18)	C11—H11C	0.96
C5—C6	1.398 (2)		
			
C2—O1—H1	112 (3)	O10—C6—C5	114.96 (13)
O1—C2—C3	110.07 (15)	C7—C6—C5	119.54 (14)
O1—C2—H2A	109.6	C6—C7—C8	119.66 (16)
C3—C2—H2A	109.6	C6—C7—H7	120.2
O1—C2—H2B	109.6	C8—C7—H7	120.2
C3—C2—H2B	109.6	C3—C8—C7	121.29 (14)
H2A—C2—H2B	108.2	C3—C8—H8	119.4
C8—C3—C4	118.80 (14)	C7—C8—H8	119.4
C8—C3—C2	121.06 (14)	C5—O9—H9	116 (2)
C4—C3—C2	120.12 (15)	C6—O10—C11	117.34 (13)
C5—C4—C3	120.48 (15)	O10—C11—H11A	109.5
C5—C4—H4	119.8	O10—C11—H11B	109.5
C3—C4—H4	119.8	H11A—C11—H11B	109.5
O9—C5—C4	122.81 (14)	O10—C11—H11C	109.5
O9—C5—C6	116.99 (13)	H11A—C11—H11C	109.5
C4—C5—C6	120.20 (13)	H11B—C11—H11C	109.5
O10—C6—C7	125.50 (15)		

### Hydrogen-bond geometry (Å, º)

**Table:**

*D*—H···*A*	*D*—H	H···*A*	*D*···*A*	*D*—H···*A*
O1—H1···O9^i^	0.74 (4)	2.11 (4)	2.773 (2)	150 (4)
O1—H1···O10^i^	0.74 (4)	2.54 (4)	3.152 (2)	142 (4)
O9—H9···O1^ii^	0.88 (4)	1.78 (4)	2.641 (2)	163 (3)

Symmetry codes: (i) *x*, −*y*+1/2, *z*+1/2; (ii) −*x*+1, *y*−1/2, −*z*+3/2.

## References

[bb2] Ashraf, Z., Rafiq, M., Seo, S. Y., Babar, M. M. & Zaidi, N. S. S. (2014). *J. Enzyme Inhib. Med. Chem.* pp. 1–10. 10.3109/14756366.2014.979346 25643758

[bb3] Ashraf, Z., Rafiq, M., Seo, S. Y., Kwon, K. S., Babar, M. M. & Zaidi, N. U. S. (2015). *Eur. J. Med. Chem.* **98**, 203–211. 10.1016/j.ejmech.2015.05.031 26025140

[bb4] Bruker (2002). *SAINT* and *SMART*. Bruker AXS Inc., Madison, Wisconsin, USA.

[bb5] Coimbra, E. S., de Almeida, M. V., JÃ°nior, C. O. R., Taveira, A. F., da Costa, C. F., de Almeida, A. C., Reis, E. F. C. & da Silva, A. D. (2010). *Chem. Biol. Drug Des.* **75**, 233–235. 10.1111/j.1747-0285.2009.00923.x 20028395

[bb6] Córdova, I., León, L. G., León, F., San Andrés, L., Luis, J. G. & Padrón, J. M. (2006). *Eur. J. Med. Chem.* **41**, 1327–1332. 10.1016/j.ejmech.2006.06.001 16828933

[bb7] Farrugia, L. J. (2012). *J. Appl. Cryst.* **45**, 849–854.

[bb8] Hans, R. H., Gut, J., Rosenthal, P. J. & Chibale, K. (2010). *Bioorg. Med. Chem. Lett.* **20**, 2234–2237. 10.1016/j.bmcl.2010.02.017 20206517

[bb1] Matteelli, A., Carvalho, A. C., Dooley, K. E. & Kritski, A. (2010). *Future Microbiol.* 5, 849–858. 10.2217/fmb.10.50 PMC2921705 20521931

[bb9] Patrick, G. L. (2001). In *An Introduction to Medicinal Chemistry*. Oxford: University Press.

[bb10] Rodríguez-Barrios, F. & Gago, F. (2004). *Curr. Top. Med. Chem.* **4**, 991–1007. 10.2174/1568026043388529 15134553

[bb11] Sheldrick, G. M. (2008). *Acta Cryst.* A**64**, 112–122. 10.1107/S0108767307043930 18156677

[bb12] Sheldrick, G. M. (2015). *Acta Cryst.* C**71**, 3–8.

[bb13] Wu, X., Öhrngren, P., Ekegren, J. K., Unge, J., Unge, T., Wallberg, H., Samuelsson, B., Hallberg, A. & Larhed, M. (2008). *J. Med. Chem.* **51**, 1053–1057. 10.1021/jm070680h 18215014

[bb14] Yasohara, Y., Miyamoto, K., Kizaki, N. & Hasegawa, J. (2001). *Tetrahedron Lett.* **42**, 3331–3333.

