# Supplementary figures and images for: Crystal structure of 5-hy­droxy­methyl-2-meth­oxy­phenol
Source: Acta Crystallogr E Crystallogr Commun. 2015 Jul 4;71(Pt 8):o540–1. doi: 10.1107/S205698901501230X (PMC4571386; doi:10.1107/S205698901501230X)

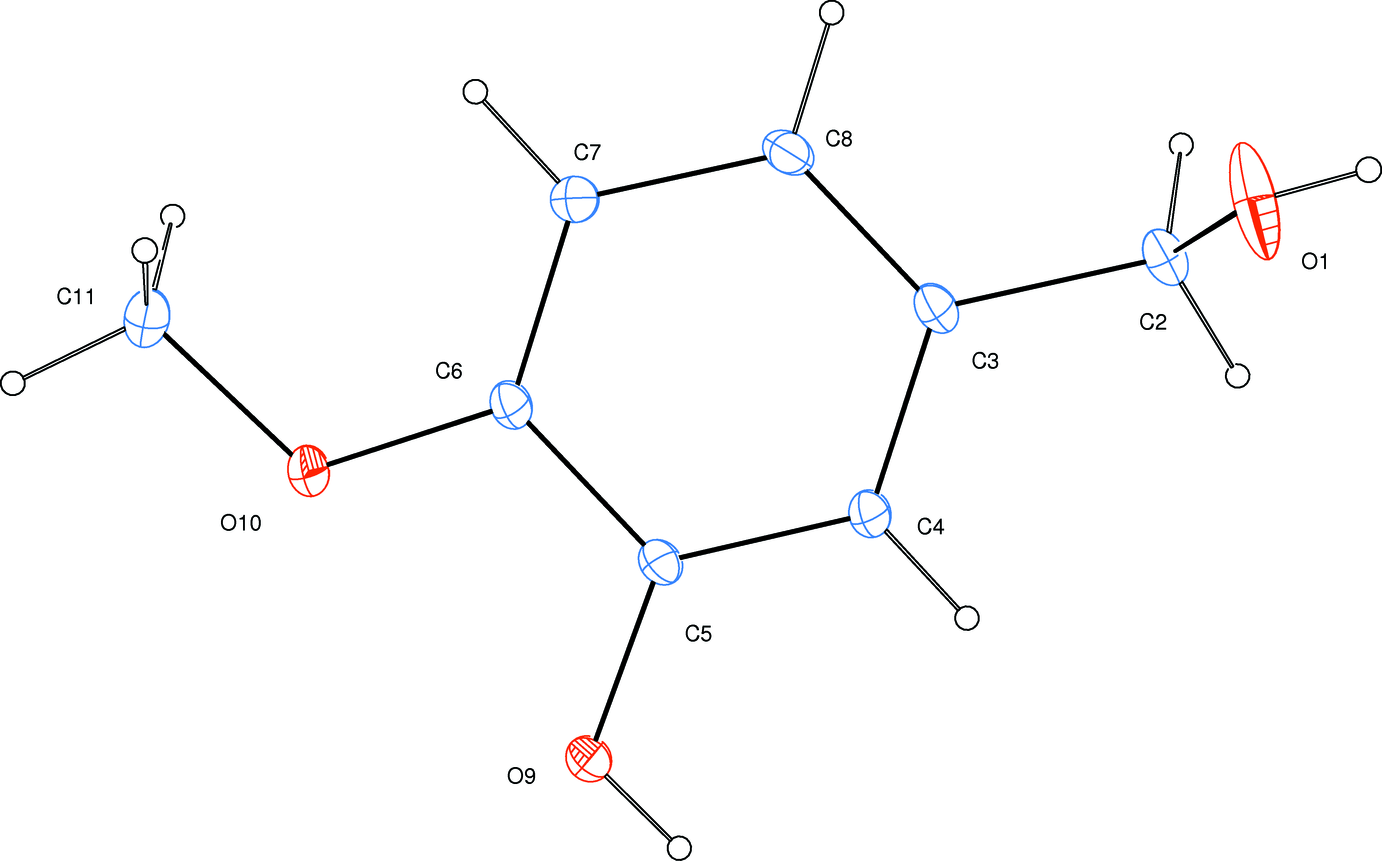

Supplement: Supplementary file 4 [file e-71-0o540-fig1.tif]

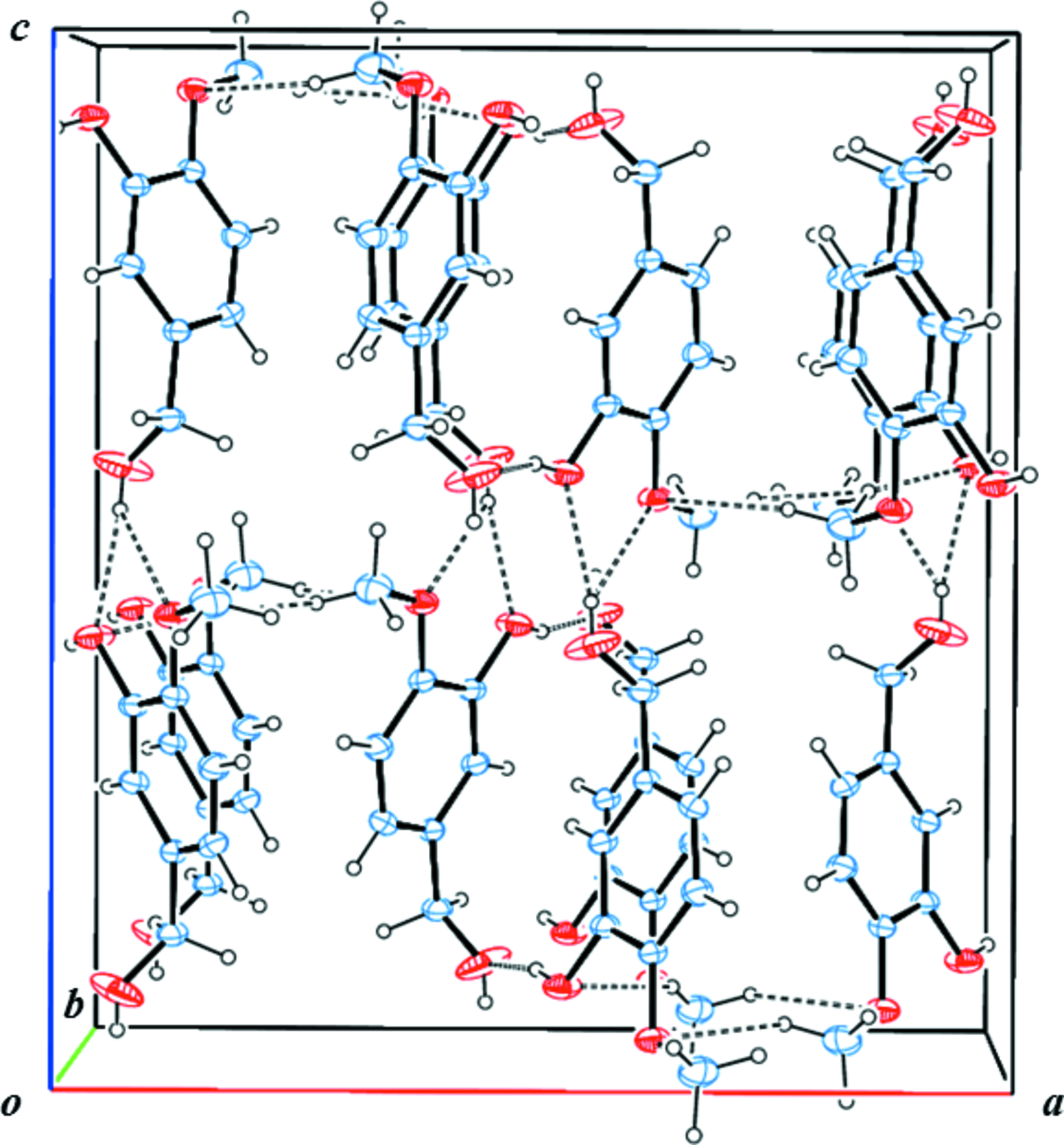

Supplement: Supplementary file 5 [file e-71-0o540-fig2.tif]
